# Idiopathic Fibrillary Glomerulonephritis: A Case Report Highlighting Diagnostic and Management Challenges

**DOI:** 10.7759/cureus.87135

**Published:** 2025-07-01

**Authors:** Emin Gayibov, Isha Gupta

**Affiliations:** 1 Third Faculty of Medicine, Charles University, Prague, CZE; 2 Department of General Surgery, University Hospital Královské Vinohrady, Prague, CZE; 3 Department of Nephrology, Garnet Health Medical Center, Middletown, USA; 4 Department of Nephrology, Middletown Medical, Middletown, USA

**Keywords:** fibrillary glomerulonephritis, glomerular disease, idiopathic fibrillary glomerulonephritis, kappa light chain, mesangial proliferation

## Abstract

Fibrillary glomerulonephritis (FGN) is a rare glomerular disease characterized by non-branching fibrils within the glomerular basement membrane, often leading to progressive renal dysfunction. Despite advances in diagnostic methods, including DNA-J heat shock protein family member B9 (DNAJB9) immunostaining, the pathogenesis and optimal treatment strategies remain poorly defined.

We present the case of a 55-year-old woman with longstanding microscopic hematuria and subnephrotic proteinuria who was diagnosed with idiopathic fibrillary glomerulonephritis (IFGN) following a renal biopsy. Histopathology revealed mesangial proliferation, thickened basement membranes, and focal crescent formation. Immunofluorescence microscopy demonstrated IgG positivity with kappa light chain restriction, while electron microscopy confirmed fibrillary deposits measuring 22 nm in diameter. DNAJB9 immunostaining was strongly positive, confirming the diagnosis. In the absence of an identifiable secondary cause, this case represents a rare instance of IFGN. The patient was managed with nephroprotective strategies, including renin-angiotensin system blockade, glycemic control, and lipid management.

This case underscores the diagnostic and therapeutic challenges associated with IFGN and highlights the importance of early recognition, histopathological confirmation, and supportive management. Further research is essential to improve prognostic assessment and develop evidence-based treatments for this rare and progressive glomerular disease.

## Introduction

Fibrillary glomerulonephritis (FGN), a rare form of glomerulonephritis first described in 1977 [1], is characterized by the presence of randomly arranged, non-branching fibrils with a diameter ranging from 15 to 25 nanometers, averaging 20 nanometers, when examined under an electron microscope [2]. It is encountered in approximately 0.5% to 1% of native kidney biopsies [3]. The renal prognosis of FGN is generally poor, with nearly 50% of patients progressing to end-stage renal disease (ESRD) within four years of diagnosis [4]. While most reported cases of FGN are sporadic, rare familial forms have been described [5]. In our case, there was no family history of glomerular disease or proteinuria suggestive of a familial pattern. The diagnosis was thus considered sporadic and idiopathic.

The diagnosis of FGN is based on several key histopathological features [6]. These include mesangial and frequently endocapillary proliferation, as well as positive immunofluorescence for polyclonal immunoglobulin G and complement component 3 deposition [6]. Electron microscopy reveals the presence of randomly arranged fibrils, measuring 12 to 22 nanometers in diameter, primarily within the mesangium and occasionally extending into the glomerular basement membrane [6]. Importantly, these fibrils do not stain with Congo red, distinguishing FGN from amyloidosis [6]. A significant recent advancement in understanding the pathogenesis of FGN has been the discovery that a major component of these fibrils is DNA-J heat shock protein family member B9 (DNAJB9). Immunohistochemical staining for this protein now allows for the diagnosis of FGN even in the absence of ultrastructural evaluation [2]. The clinical presentation of FGN may include fatigue, edema, proteinuria, hematuria, nephrotic syndrome, renal insufficiency, and hypertension [4].

Although many previously reported cases of FGN were considered idiopathic and occurred without any associated systemic disease [7], it is now recognized that FGN often represents a secondary glomerular disease process. It has been linked to autoimmune diseases, infections, and malignancies in a significant proportion of cases [8]. As a result, truly idiopathic fibrillary glomerulonephritis (IFGN), without any identifiable underlying disease, appears to be relatively uncommon, accounting for less than half of all diagnoses [4].

This case report presents the clinical course of a 55-year-old female patient diagnosed with IFGN. It highlights the challenges in managing this rare condition and underscores the need for further research in prognostic evaluation, potential biomarkers, and early treatment strategies. Notably, she had no history of commonly associated systemic conditions, emphasizing the rare but severe nature of IFGN. This case also underscores the critical need for continued research to improve patient outcomes in this rare disease entity.

## Case presentation

A 55-year-old woman with progressive renal dysfunction was evaluated following a prolonged history of microscopic hematuria, subnephrotic proteinuria, and albuminuria. Her serum creatinine was 1.1 mg/dL, and her protein-creatinine ratio was 0.84. Comprehensive autoimmune and infectious serologic testing, including antinuclear antibodies, double-stranded DNA antibodies, rheumatoid factor, hepatitis panel, human immunodeficiency virus, and antineutrophil cytoplasmic antibodies - was negative. Complement component 3 and 4 levels were within normals ranges. Hemoglobin A1c was 5.9%, and serum and urine protein electrophoresis revealed no evidence of monoclonal gammopathy. No features suggestive of dysproteinemia were identified on repeated follow-up assessments. Immunofixation electrophoresis was not performed as there were no clinical or laboratory indicators warranting further evaluation for monoclonal gammopathy. A summary of the patient's laboratory findings is presented in Table 1.

**Table 1:** A summary of the patient's laboratory findings Normal reference ranges were obtained from Express Lab at Garnet Health Medical Center [9]. C3: complement component 3; C4: complement component 4, mg/dL: milligrams per deciliter

Parameter	Result	Normal reference range for female adults
Serum creatinine	1.1 mg/dL	0.6-1.2 mg/dL
Protein-creatinine ratio	0.84	<0.2
Antinuclear antibody	Negative	-
Double-stranded DNA antibodies	Negative	-
C3	120 mg/dL	90-180 mg/dL
C4	30 mg/dL	10-40 mg/dL
Rheumatoid factor	Negative	-
Hepatitis panel	Negative	-
Human immunodeficiency virus	Negative	-
Antineutrophil cytoplasmic antibodies	Negative	-
Hemoglobin A1c	5.9%	<5.7%
Serum protein electrophoresis	No monoclonal bands	-
Urine protein electrophoresis	No monoclonal bands	-

Retrospective chart review revealed that microscopic hematuria had been present on multiple serial urinalyses for several years prior to presentation, initially identified during evaluation for unrelated flank discomfort. Hematuria persisted without accompanying red blood cell casts or urinary tract infections. Subnephrotic proteinuria developed subsequently, with repeated urine protein-creatinine ratios ranging between 0.3 and 0.7. Baseline serum creatinine remained between 0.9 and 1.1 mg/dL, and estimated glomerular filtration rate (eGFR) was consistently above 60 mL/min/1.73 m² until recent decline. No nephrotic-range proteinuria or gross hematuria was noted during this earlier period.

The patient’s medical history was significant for hypertension, obesity, type 2 diabetes mellitus, hyperlipidemia, hyperuricemia, and gastroesophageal reflux disease. She was on long-term treatment with statin, verapamil, omeprazole, metformin, lisinopril, and allopurinol. Notably, her father had a history of kidney failure requiring nephrectomy at a young age due to a presumed kidney infection.

**Figure 1:**
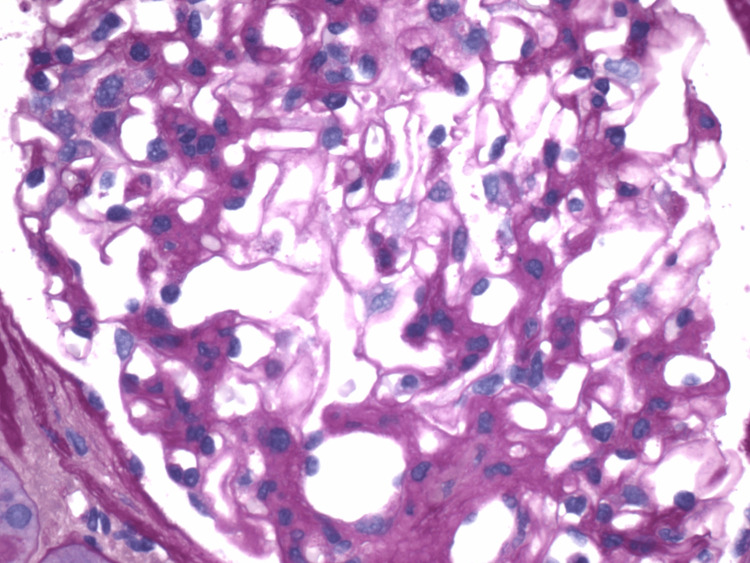
Renal biopsy demonstrating mesangial expansion, stained with hematoxylin and eosin Marked mesangial matrix expansion accompanied by increased mesangial cellularity. Glomerular capillary loops appear thickened and are partially obscured by the expanded mesangial areas.

**Figure 2:**
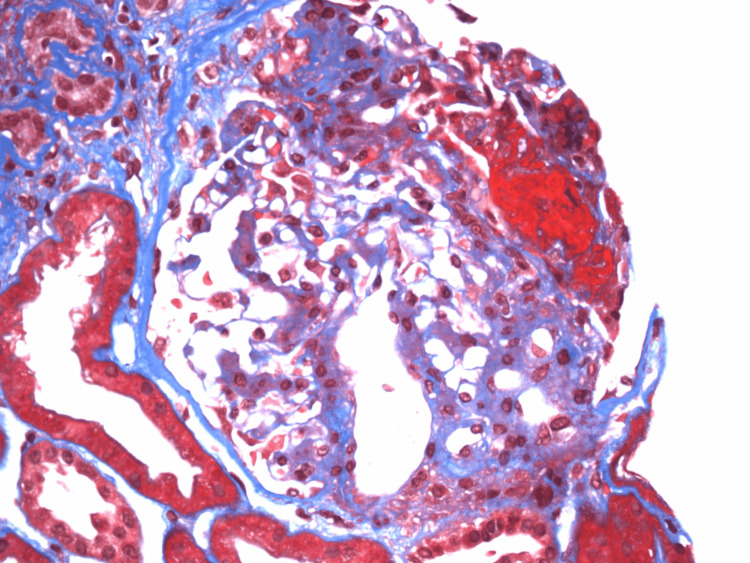
Renal biopsy demonstrating a necrotizing crescent, stained with Masson’s trichrome Masson’s trichrome stain highlighting a necrotizing crescent involving the glomerular tuft. The area of fibrinoid necrosis appears intensely red within Bowman’s space, surrounded by proliferating crescentic epithelial cells. Blue staining delineates interstitial collagen deposition and early tubular basement membrane fibrosis, consistent with ongoing glomerular injury.

**Figure 3:**
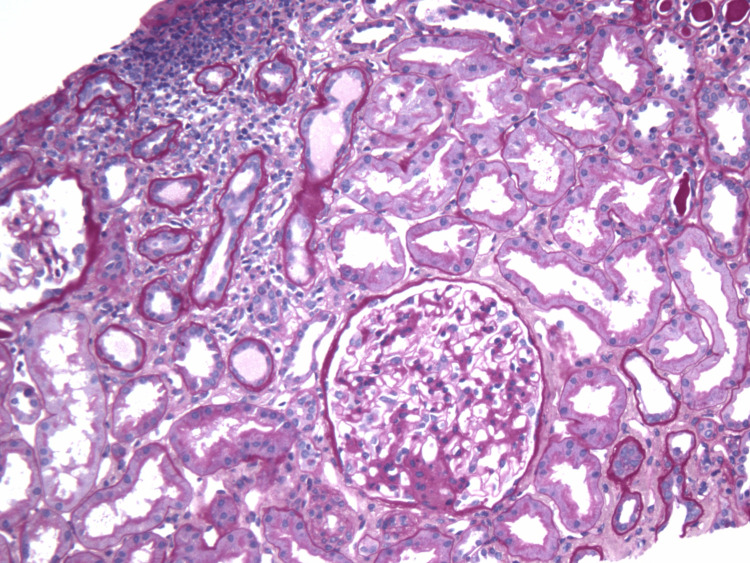
Renal biopsy demonstrating interstitial inflammation, stained with hematoxylin and eosin Low-power hematoxylin and eosin stain showing patchy interstitial inflammation characterized by dense mononuclear cell infiltrates, particularly in the upper cortex. Tubules are mildly atrophic, and the surrounding interstitium is expanded.

A renal biopsy revealed 26 glomeruli, of which three demonstrated global sclerosis. One glomerulus showed a necrotizing crescent, and another had a fibrocellular crescent. The remaining glomeruli exhibited diffuse mesangial hypercellularity and thickened basement membranes. Minimal interstitial inflammation (<10%), along with tubular atrophy, interstitial fibrosis, and mild intimal fibrosis of the renal arteries, was observed. Representative histopathological images of the renal biopsy are shown in Figures 1-3, highlighting the observed glomerular abnormalities, including mesangial expansion, necrotizing crescent formation, and interstitial inflammation.

Immunofluorescence microscopy demonstrated IgG positivity, including subclasses IgG2, IgG3, and IgG4, as well as kappa light chain restriction. C3 staining was also present. However, staining for lambda light chain, IgA, IgM, and C1q was negative, ruling out immune complex-mediated and complement-mediated glomerulonephritides. While most cases of FGN demonstrate IgG1 and IgG4 subclass staining, with IgG4 typically more intense [10], our case revealed positive staining for IgG2, IgG3, and IgG4, deviating from this common profile. This discordance highlights the heterogeneity of IgG subclass expression in FGN and underscores the importance of integrating immunofluorescence results with systemic evaluations before diagnosing monoclonal gammopathy-associated glomerulopathy.

**Figure 4:**
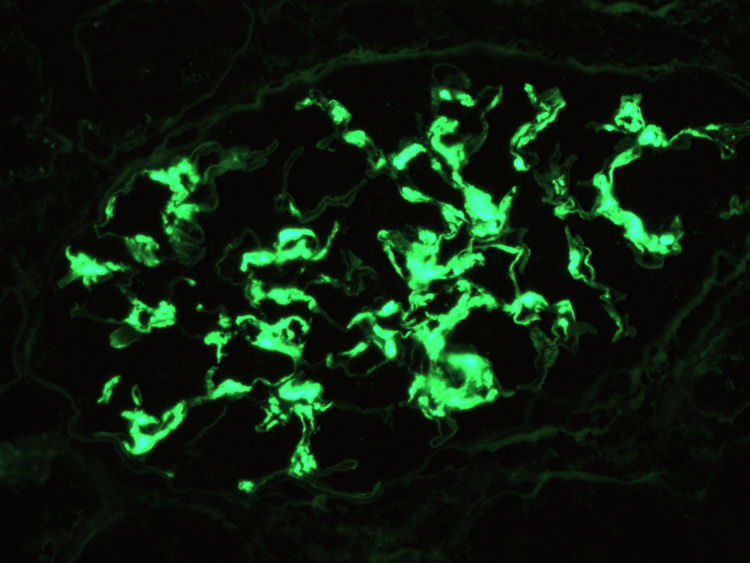
Immunofluorescence microscopy showing granular IgG deposition Bright granular IgG deposition along the glomerular capillary loops and within the mesangium. This staining pattern is characteristic of fibrillary glomerulonephritis. The absence of IgA, IgM, C1q, and lambda light chain staining supports a non–immune complex-mediated process with kappa light chain restriction.

**Figure 5:**
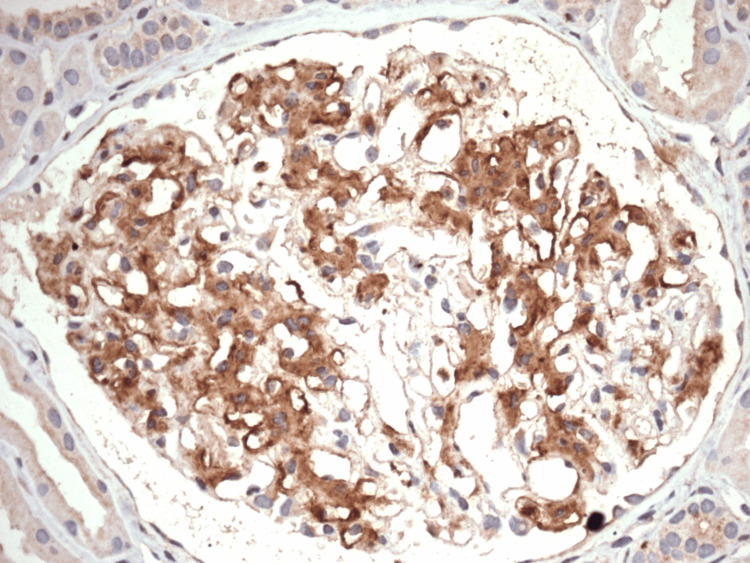
A positive confirmatory DNA-J heat shock protein family member B9 immunostain Immunohistochemical staining for DNA-J heat shock protein family member B9 shows diffuse, strong granular positivity within the glomerular tufts. This staining pattern is diagnostic of fibrillary glomerulonephritis, confirming the presence of DNAJB9-containing fibrils. No background staining is seen in the tubules or interstitium.

A confirmatory DNAJB9 immunostain was strongly positive, confirming the diagnosis of FGN. Immunofluorescence findings are illustrated in Figures 4-5, demonstrating positive staining for IgG and DNAJB9, respectively.

**Figure 6:**
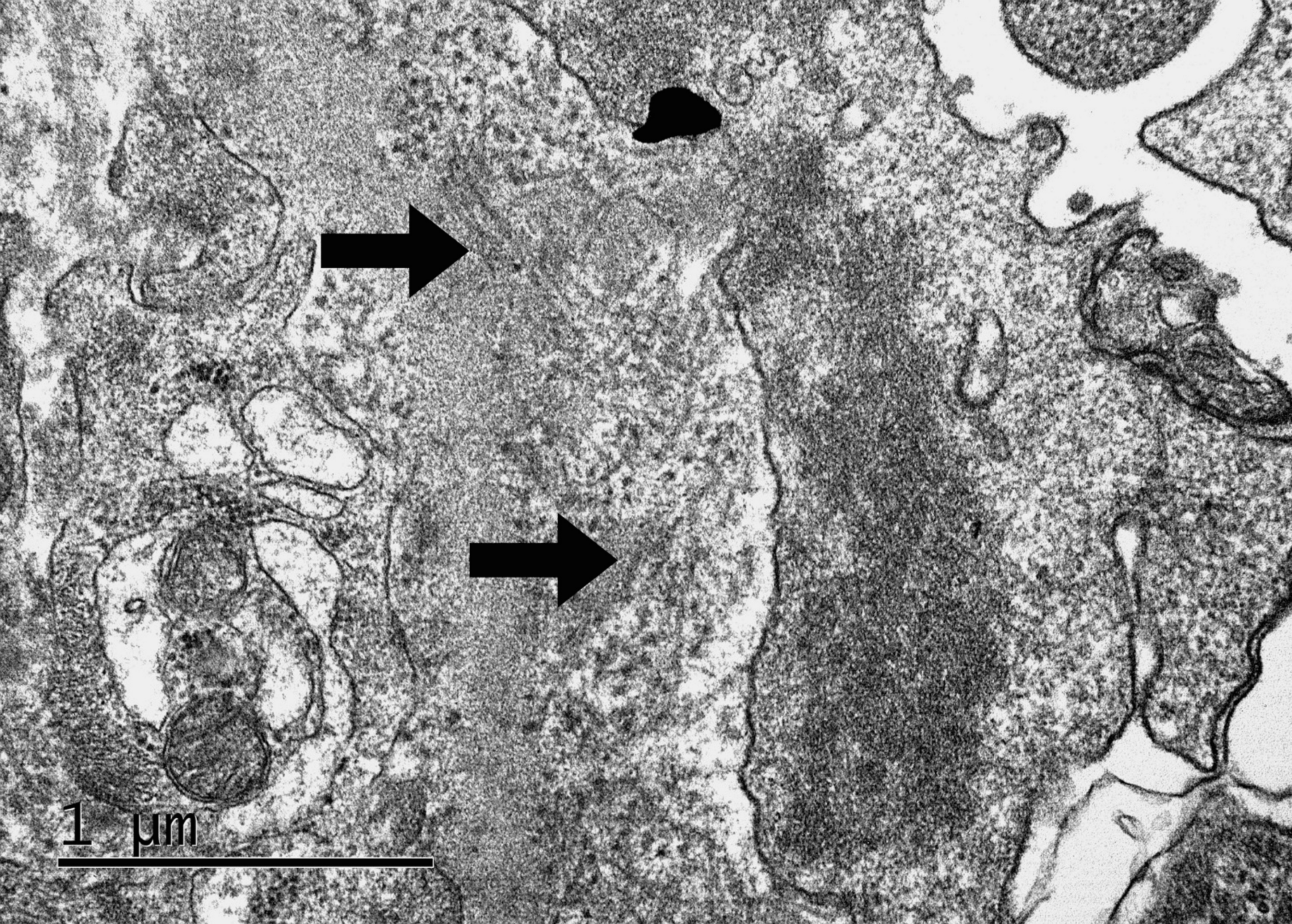
Electron microscopy revealing fibrillary glomerular deposits in fibrillary glomerulonephritis Randomly arranged, non-branching fibrillary deposits (indicated by arrows) are seen within the expanded mesangial matrix and along the glomerular basement membrane. The fibrils measure approximately 22 nm in diameter and lack the periodicity characteristics of amyloid. No immune-type electron-dense deposits or extraglomerular fibrillary material are observed.

Electron microscopy revealed mesangial expansion with matrix accumulation and the presence of randomly arranged, non-branching fibrillary deposits measuring 22 nm in diameter. No immune-type electron-dense deposits or extraglomerular fibrillary deposits were observed. Importantly, no histologic features suggestive of diabetic nephropathy were identified, supporting the primary glomerular nature of the disease rather than a secondary process related to diabetes. Ultrastructural findings on electron microscopy are shown in Figure 6.

The patient’s FGN was characterized by diffuse mesangial proliferative changes, focal necrotizing and crescentic features, and mild arteriosclerosis. Given the known poor prognosis associated with this condition, close nephrology follow-up was arranged to monitor renal function and progression of proteinuria. The patient was advised on renin-angiotensin system blockade with lisinopril and to implement lifestyle modifications to mitigate cardiovascular and renal risks.

Management was primarily conservative, focusing on nephroprotection due to the absence of standardized treatment protocols and the variable response to immunosuppressive therapy. Blood pressure control was prioritized through the use of lisinopril, an angiotensin-converting enzyme inhibitor, to reduce proteinuria and slow disease progression by mitigating glomerular hyperfiltration. The target blood pressure was set at less than 130/80 mmHg. The patient was maintained on lisinopril 20 mg daily throughout the early disease course, during which renal function remained stable with eGFR >45 mL/min/1.73m². Given the potential benefits of higher dosing, up-titration toward the maximum recommended dose, 40 mg daily, was considered. However, as kidney function began to decline progressively, lisinopril was temporarily held during episodes of acute hemodynamic instability or serum creatinine rise >30% from baseline, and later reintroduced at a reduced dose of 10 mg daily under close monitoring. This approach was in accordance with nephrology recommendations to balance renoprotective effects with safety in the setting of progressive chronic kidney disease (CKD).

Glycemic control remained stable, with an HbA1c of 5.9%, allowing continued use of metformin, with dose adjustments as needed based on kidney function. As eGFR declined (<45 mL/min/1.73m²), the metformin dose was reduced from 1000 mg to 500 mg twice daily to ensure safety and minimize the risk of lactic acidosis. A sodium-glucose co-transporter 2 (SGLT-2) inhibitor, specifically empagliflozin, was initiated to provide additional nephroprotection and cardiovascular benefit, based on emerging evidence supporting its use in patients with CKD, including glomerular diseases such as FGN. Recent studies have demonstrated that SGLT2 inhibitors can significantly reduce nephrotic-range proteinuria even in non-diabetic individuals. In addition to slowing the decline in eGFR, these agents have been shown to delay the onset of ESRD, mitigate renal inflammation, lessen interstitial fibrosis, and contribute to overall improvement in kidney function [11].

Hyperlipidemia was managed with statin therapy due to the patient’s elevated cardiovascular risk, with close monitoring for potential statin-associated rhabdomyolysis, as patients with CKD are at increased risk. Atorvastatin was selected for lipid management due to its well-established cardiovascular benefits in patients with CKD and a more favorable renal safety profile compared to rosuvastatin. Recent pharmacoepidemiologic data have shown that rosuvastatin is associated with a higher incidence of hematuria, proteinuria, and progression to kidney failure in patients with pre-existing CKD [12]. Allopurinol was continued to prevent hyperuricemia-related nephropathy, considering the patient’s history of hyperuricemia.

Immunosuppressive therapy was not initiated at the time of diagnosis, as histologic findings indicated focal active lesions, e.g., necrotizing and fibrocellular crescents, but limited chronic damage. However, future treatment with rituximab, cyclophosphamide, or corticosteroids was considered in the event of worsening proteinuria exceeding 3g/day or a significant decline in renal function, defined as a reduction in eGFR of more than 40%. Regular nephrology follow-ups were scheduled every three to six months for serial monitoring of serum creatinine, proteinuria, and albuminuria, with annual kidney function reassessments, including urinalysis and imaging if indicated. Despite preserved renal function at the time of diagnosis, the progressive nature of FGN poses a high risk of progression to ESRD within five to ten years, necessitating early planning for potential renal replacement therapy. The patient was counseled on the possibility of requiring dialysis or kidney transplantation in the future. A structured approach to the management of FGN is summarized in Table 2, detailing key aspects of nephroprotection, metabolic control, and potential future interventions.

**Table 2:** Multidisciplinary management approach in a patient with idiopathic fibrillary glomerulonephritis Interventions were tailored to optimize renal protection, metabolic control, and cardiovascular risk reduction. Future immunosuppressive therapy was reserved for disease progression, defined by increasing proteinuria or declining renal function. Close nephrology follow-up and patient education were emphasized due to the high risk of progression to end-stage renal disease.

Management aspect	Treatment/intervention	Rationale
Blood pressure control	lisinopril	reduce proteinuria, slow chronic kidney disease progression, target blood pressure <130/80 mmHg
Glycemic control	metformin, dose-adjusted	maintain HbA1c <6.5%, prevent further renal deterioration
Nephroprotection	empagliflozin	provide additional renal and cardiovascular protection
Lipid management	statin therapy	reduce cardiovascular risk, monitor for rhabdomyolysis
Uric acid management	allopurinol	prevent hyperuricemia-related nephropathy
Immunosuppression consideration	deferred at diagnosis; potential future use of rituximab, cyclophosphamide, or corticosteroids	initiate if proteinuria exceeds >3g/day or estimated glomerular filtration rate declines by >30%
Long-term monitoring	nephrology follow-up every 3–6 months, serial monitoring of creatinine, proteinuria, and albuminuria	assess disease progression and guide therapeutic adjustments
Prognosis planning	patient counseling on potential end-stage renal disease and renal replacement therapy	high risk of progression to end-stage renal disease within 5–10 years

Over the subsequent years, the patient’s renal function gradually declined despite adherence to nephroprotective therapy. Serial serum creatinine measurements over a five-year period showed gradual deterioration in renal function from 1.1 mg/dL to over 2.2 mg/dL, an eGFR decline to below 30 mL/min/1.73 m² and a progression from stage 3 to stage 4 CKD. Urinalysis consistently revealed microscopic hematuria and progressive proteinuria, which eventually reached the nephrotic range. However, urine microscopy did not report dysmorphic red blood cells or red blood cell casts. Immunosuppressive therapy was deferred as histologic activity was limited, and the potential risks were judged to outweigh the anticipated benefits in the context of advancing chronic kidney disease. Despite aggressive risk factor management, the natural history of IFGN led to irreversible renal deterioration over approximately five years from diagnosis.

This case highlights an incidentally diagnosed IFGN in a patient with longstanding microscopic hematuria and subnephrotic proteinuria, confirmed by the presence of glomerular fibrillary deposits, DNAJB9 positivity, and the absence of monoclonal gammopathy. Notably, immunofluorescence demonstrated kappa light chain restriction, which can raise concern for an underlying monoclonal gammopathy. However, multiple myeloma was excluded based on normal serum and urine protein electrophoresis, absence of monoclonal bands, and unremarkable serum calcium, hemoglobin, and creatinine trends. There was no bone pain, lytic lesions, or clinical signs suggestive of plasma cell dyscrasia. These findings support a non-monoclonal etiology. Prior studies have shown that light chain restriction may be present in approximately 5% of FGN cases without associated monoclonal gammopathy, reflecting the potentially misleading nature of this finding in the absence of confirmatory systemic features [3].

To evaluate for other malignancies as a potential secondary cause of fibrillary glomerulonephritis, the patient underwent age-appropriate cancer screening. A screening colonoscopy performed was normal. Serial computed tomography scans of the abdomen and pelvis showed no evidence of mass lesions, lymphadenopathy, or organomegaly. A computed tomography scan of the brain was also unremarkable. Laboratory data, including complete blood count, lactate dehydrogenase, liver function tests, and urinalysis, revealed no abnormalities suggestive of underlying malignancy. No clinical signs or symptoms of malignancy were reported during longitudinal follow-up.

Given the uncertain disease trajectory and the lack of robust treatment strategies, the management approach prioritized close monitoring and nephroprotective interventions to delay progression and optimize long-term renal outcomes. Although no overt systemic disease was identified, the presence of nephrotic-range proteinuria and histologic evidence of active glomerular injury underscores the need for vigilant monitoring to delay progression to ESRD. Importantly, the absence of monoclonal proteins, negative comprehensive autoimmune and infectious serologies, and the lack of histologic features suggestive of diabetic nephropathy support the diagnosis of an idiopathic rather than secondary form of FGN. Based on these findings, the patient requires long-term nephrology follow-up, aggressive management of hypertension and proteinuria, and renal replacement therapy.

## Discussion

FGN is an exceptionally rare glomerular disease characterized by the deposition of randomly arranged, non-branching fibrils within the glomerular basement membrane. Definitive diagnosis is established through electron microscopy and immunohistochemical staining for DNAJB9, a specific biomarker for FGN [2, 13]. This case highlights a unique presentation of IFGN in a patient with no apparent secondary etiology, thereby contributing to the limited literature on this condition.

Histopathological examination plays a crucial role in distinguishing FGN from other fibrillary and immune complex-mediated glomerulopathies, such as amyloidosis and diabetic nephropathy. Unlike amyloid deposits, FGN lacks Congo red positivity and demonstrates a distinct immunofluorescence pattern [10]. However, a recently described variant known as congophilic FGN can show Congo red positivity despite lacking the proteomic signature of amyloid, further emphasizing the need for DNAJB9 immunohistochemistry and mass spectrometry in challenging cases [14]. Additionally, while diabetic patients often show mesangial expansion and nodular sclerosis, the presence of fibrillary deposits and DNAJB9 positivity confirms the diagnosis of FGN [10]. The presence of crescents, as noted in our case, has been reported in a small subset of FGN cases and is believed to indicate a more aggressive disease course [15]. The pathogenesis of IFGN remains unclear, though it is hypothesized to be immune-mediated, given the frequent presence of immune complexes and complement activation [16]. While previous studies have associated FGN with autoimmune diseases, infections, and malignancies, extensive evaluation in this case failed to identify any underlying condition, supporting the diagnosis of IFGN.

DNAJB9 is a heat shock protein co-chaperone primarily localized in the endoplasmic reticulum and is normally intracellular. In fibrillary glomerulonephritis, it abnormally accumulates extracellularly in the glomeruli, forming homogeneous deposits in the mesangium and capillary walls [11]. Immunostaining for DNAJB9 has demonstrated a sensitivity of 98% and a specificity of 99% in the diagnosis of FGN [13]. While DNAJB9 serves as a highly specific and sensitive diagnostic biomarker, recent evidence also suggests its potential pathogenic involvement. The protein frequently coexists with IgG and complement components in glomerular deposits, suggesting a role in autoimmune-mediated injury. Although its exact function in disease development remains unclear, these findings imply that DNAJB9 may participate in the immunopathogenesis of FGN in addition to its diagnostic utility [11].

Treatment of FGN remains challenging due to the absence of standardized guidelines. Current management strategies emphasize nephroprotection with renin-angiotensin system inhibitors and supportive care. Immunosuppressive therapies, including corticosteroids, rituximab, and cyclophosphamide, have been attempted in select cases with variable success [17-19]. Despite these interventions, FGN frequently progresses to ESRD, necessitating long-term nephrology follow-up and eventual consideration of renal replacement therapy [3].

This case underscores the importance of early recognition of FGN in patients presenting with unexplained proteinuria and hematuria. DNAJB9 immunostaining has emerged as a pivotal diagnostic tool, enabling differentiation from other glomerular diseases with similar morphological features [13]. Further research is needed to elucidate the pathophysiology of FGN, identify reliable prognostic markers, and develop effective therapeutic approaches for this rare condition.

## Conclusions

FGN is a rare and poorly understood glomerular disease with a high risk of progression to ESRD. This case highlights an idiopathic presentation with crescent formation, underscoring the importance of vigilant monitoring and nephroprotective strategiens. DNAJB9 immunostaining has emerged as a critical diagnostic tool, allowing reliable differentiation from other glomerular pathologies. Given the lack of standardized treatment, early recognition and risk factor management remain essential to delaying disease progression. Further research is needed to improve prognostic assessment and to develop effective therapeutic approaches for this challenging condition.
